# Entre incertezas e mundos possíveis: as políticas da linguagem dos riscos

**DOI:** 10.1590/0102-311XPT256625

**Published:** 2026-04-27

**Authors:** Guilherme José Parisi, Pedro Paulo Gomes Pereira

**Affiliations:** 1 Escola Paulista de Medicina, Universidade Federal de São Paulo, São Paulo, Brasil.

Mary Jane Spink tem se afirmado como uma das principais referências na compreensão psicossocial dos riscos no Brasil, contribuindo decisivamente para deslocar o tema de leituras estritamente probabilísticas para abordagens que consideram linguagem, práticas e contextos. Em *Usos da Linguagem dos Riscos: Das Certezas Passadas às Imponderabilidades Contemporâneas* ^1^, publicado pela Editora Fiocruz, a autora consolida um percurso teórico-metodológico singular ao articular construcionismo social, análise discursiva e debates centrais da Saúde Coletiva, compondo um quadro interpretativo sensível às relações de poder, às formas de vida e às racionalidades que atravessam a produção do risco. O livro não apenas revisita tradições consolidadas, mas reposiciona a própria noção de risco como um campo de disputas simbólicas, morais e materiais, evidenciando como sujeitos, instituições, dispositivos e artefatos co-produzem, estabilizam e reconfiguram sentidos no cotidiano. Ao fazê-lo, ilumina a linguagem dos riscos como prática socialmente situada, inseparável dos arranjos sociotécnicos, dos regimes de verdade e das dinâmicas de governança que moldam a vida coletiva.

A obra estrutura-se em quatro partes que, mais do que historicizar transformações conceituais, tornam visíveis os regimes de verdade que sustentam diferentes formas de imaginar, medir e governar o risco. Na primeira parte, Spink reconstrói a genealogia do conceito, elucidando como a quantificação, os seguros marítimos e a emergência da probabilidade constituíram uma gramática moderna do risco - uma gramática que naturaliza operações de cálculo, previsão e responsabilização. Ao tensionar essa hegemonia objetivista, a autora evidencia que definir risco é sempre um trabalho situado, atravessado por interesses, valores e disputas epistemológicas, questão fundamental para a Saúde Coletiva, frequentemente permeada por imperativos tecnocráticos e modelos normativos de racionalidade.

A segunda parte desloca o olhar ao explorar a positividade do risco, concebido não apenas como ameaça, mas como experiência estética, sensorial e identitária. Ao abordar jogos, *flow* e esportes radicais, Spink ilumina dimensões amplamente negligenciadas na literatura brasileira, trazendo à cena práticas de risco deliberado que produzem pertencimento, intensidade e modos específicos de subjetivação. Essa inflexão complexifica a visão dominante, que tende a reduzir o risco ao eixo prevenção-dano-patologia, e abre caminho para entender como emoções, prazeres e buscas por vitalidade compõem ecologias morais e afetivas pouco consideradas nos estudos de saúde.

A terceira parte, centrada na governamentalidade, retoma o risco como tecnologia de gestão populacional. Ao articular produção de dados, vigilância, seguros e discursos sobre estilo de vida, Spink revela como o risco opera como instrumento de governo e, simultaneamente, como linguagem que convoca sujeitos a gerenciarem suas próprias condutas. Os capítulos sobre tabagismo e sobre a corrida pela vacina - especialmente no contexto pandêmico - traduzem com nitidez como discursos de ameaça, culpa, confiança e responsabilidade circulam e se sedimentam, conformando adesões, resistências e hesitações. A análise da hesitação vacinal, ancorada em pesquisas da autora, mostra continuidades entre epidemias, destacando que o risco não é apenas representado, mas encarnado em relações de confiança, memórias sociais e experiências corporificadas de vulnerabilidade.

Na quarta e última parte, Spink volta-se às incertezas da era genômica e às transformações ambientais do Antropoceno. Ao mobilizar debates sobre biotecnologias, identidades em risco e mudanças climáticas, explicita como a linguagem dos riscos atua tanto como ferramenta de problematização (abrindo mundos possíveis) quanto como dispositivo de governo orientado à antecipação de futuros incertos. O diálogo com questões de ética, desigualdades e negacionismo sublinha a pertinência do enfoque psicossocial para compreender controvérsias científicas e políticas que mobilizam especialistas, mídias, coletivos, algoritmos, legislações e múltiplas formas de *expertise* leiga.

O grande mérito do livro reside em recolocar o risco como construção situada, polissêmica e continuamente negociada, afastando-se de leituras que o tratam como entidade abstrata, universal ou meramente probabilística. Ao enfatizar os “usos” da linguagem dos riscos, Spink desloca o foco da essência para a prática, da substância fixa para o acontecimento relacional: o risco não preexiste às situações, mas emerge de interações concretas, de disputas discursivas, de dispositivos técnicos e de materialidades que configuram modos específicos de ver, sentir e agir no mundo. Nessa perspectiva, o risco é performado (e não apenas representado) por meio de rotinas institucionais, saberes especializados, instrumentos de medição, algoritmos, documentos, regulações, afetos e expectativas, compondo ecologias sociotécnicas em que sujeitos e objetos se co-constituem.

Esse enquadramento é particularmente fecundo para a Saúde Coletiva porque evidencia tensões estruturantes do campo: entre o objetivismo epidemiológico, que tende a conceber o risco como variável quantificável e neutra, e abordagens qualitativas e etnográficas, que revelam os modos pelos quais experiências, moralidades e relações de poder moldam o que conta como perigo; entre a prescrição normativa, orientada por imperativos de prevenção e controle, e a experiência vivida, marcada por contextos de incerteza, vulnerabilidade, agência e improvisação; entre a responsabilização individual - frequentemente inscrita em discursos de autocuidado, estilo de vida e meritocracia sanitária - e os processos sociais de produção de desigualdades, que distribuem riscos de modo assimétrico e historicamente situado.

Ao iluminar essas fricções, Spink mostra que pensar o risco exige atentar não apenas para cálculos epidemiológicos, mas também para os regimes de verdade que autorizam determinados enunciados, para as infraestruturas que sustentam práticas de governança, e para os modos como diferentes coletivos negociam sentidos, resistências e formas de habitar a incerteza.

Ainda que densa e metodologicamente consistente, a obra abre brechas produtivas para aprofundamentos futuros, especialmente no cruzamento com tradições antropológicas e sociológicas que investigam como riscos são performados, materializados e moralmente organizados. A perspectiva construcionista de Spink encontra ressonância com debates sobre co-produção entre saberes, corpos e artefatos - de Sheila Jasanoff, Bruno Latour e Annemarie Mol - que poderiam expandir ainda mais a análise para controvérsias sociotécnicas e formas de autoridade epistêmica. Paralelamente, reflexões clássicas de Ulrich Beck e Mary Douglas permitiriam tensionar fronteiras entre risco, pureza, culpa, responsabilidade e moralidades cotidianas, enquanto trabalhos de Deborah Lupton, Adriana Petryna e Veena Das contribuiriam para adensar discussões sobre afetos, temporalidades, precariedades e vulnerabilidades distribuídas. Longe de constituírem lacunas, esses pontos de contato ainda insuficientemente explorados delineiam um horizonte vibrante de articulações teóricas capazes de ampliar a potência analítica da abordagem psicossocial.

Sem pretender esgotar o tema, Spink abre caminhos promissores para investigações sobre risco-aventura - ainda incipientes no Brasil - e sobre modos emergentes de subjetivação produzidos pela governança antecipatória dos riscos. Trata-se de uma contribuição madura, rigorosa e instigante, que reafirma a centralidade das Ciências Sociais e Humanas em Saúde para compreender fenômenos que transbordam a lógica biomédica e se enraízam na vida social.

Ao final, a autora oferece ao campo não apenas um arcabouço teórico robusto, mas também uma agenda de pesquisa aberta, sensível às transformações sociotécnicas e às tensões políticas que atravessam o presente. Seu trabalho convoca leitores e pesquisadores a seguir interrogando os modos pelos quais produzimos, circulamos e politizamos a linguagem dos riscos, reconhecendo-a como prática que modela percepções, orienta intervenções e fabrica futuros possíveis. Ao fazer desse convite uma tarefa coletiva, Spink reafirma que compreender o risco é, antes de tudo, compreender os mundos que estamos incessantemente criando.
